# Exploration of Germline Correlates and Risk of Immune-Related Adverse Events in Advanced Cancer Patients Treated with Immune Checkpoint Inhibitors

**DOI:** 10.3390/curroncol31040140

**Published:** 2024-03-30

**Authors:** Emma Titmuss, Irene S. Yu, Erin D. Pleasance, Laura M. Williamson, Karen Mungall, Andrew J. Mungall, Daniel J. Renouf, Richard Moore, Steven J. M. Jones, Marco A. Marra, Janessa J. Laskin, Kerry J. Savage

**Affiliations:** 1 Department of Medical Oncology, BC Cancer, Vancouver, BC V5Z 4E6, Canada; emma.titmuss@bccancer.bc.ca (E.T.); drenouf@bccancer.bc.ca (D.J.R.); jlaskin@bccancer.bc.ca (J.J.L.); 2 Department of Medical Oncology, BC Cancer, Surrey, BC V3V 1Z2, Canada; irene.yu@bccancer.bc.ca; 3 Canada’s Michael Smith Genome Sciences Centre, Vancouver, BC V5Z 4S6, Canada; epleasance@bcgsc.ca (E.D.P.); amungall@bcgsc.ca (A.J.M.); rmoore@bcgsc.ca (R.M.); sjones@bcgsc.ca (S.J.M.J.); mmarra@bcgsc.ca (M.A.M.); 4 Pancreas Centre BC, Vancouver, BC V5Z 1G1, Canada; 5 Michael Smith Laboratories, University of British Columbia, Vancouver, BC V6T 1Z4, Canada; 6 Department of Medical Genetics, University of British Columbia, Vancouver, BC V6T 2A1, Canada

**Keywords:** immunotherapy, immune-related adverse events, genomics, cancer, immune checkpoint inhibitors

## Abstract

Immune checkpoint inhibitors (ICIs) are increasingly used in the treatment of many tumor types, and durable responses can be observed in select populations. However, patients may exhibit significant immune-related adverse events (irAEs) that may lead to morbidity. There is limited information on whether the presence of specific germline mutations may highlight those at elevated risk of irAEs. We evaluated 117 patients with metastatic solid tumors or hematologic malignancies who underwent genomic analysis through the ongoing Personalized OncoGenomics (POG) program at BC Cancer and received an ICI during their treatment history. Charts were reviewed for irAEs. Whole genome sequencing of a fresh biopsy and matched normal specimens (blood) was performed at the time of POG enrollment. Notably, we found that MHC class I alleles in the HLA-B27 family, which have been previously associated with autoimmune conditions, were associated with grade 3 hepatitis and pneumonitis (q = 0.007) in patients treated with combination PD-1/PD-L1 and CTLA-4 inhibitors, and PD-1 inhibitors in combination with IDO-1 inhibitors. These data highlight that some patients may have a genetic predisposition to developing irAEs.

## 1. Introduction

The introduction of immune checkpoint inhibitors (ICIs) has revolutionized the treatment of a broad range of cancers. Following the initial approval of ipilimumab in metastatic melanoma in 2011, multiple FDA-approved ICIs have been introduced into clinical care for a wide variety of solid and hematologic malignancies [1]. Indications are expanding rapidly every year, including tumor agnostic indications for cancers that exhibit microsatellite instability or have a high tumor mutation burden, defined as ≥10 mutations/Mb [2,3]. One recent study estimated that 43.6% of cancer patients in the United States were eligible for ICIs in 2018 compared to 1.5% in 2011 [4].

The mechanism of action of ICIs, through inhibition of molecules responsible for peripheral tolerance, can lead to a spectrum of immune-related adverse events (irAEs) that can affect any organ system [5]. Meta-analyses have shown that the most common irAEs (of any grade) following a PD-1 inhibitor were dermatologic (24.3%), gastrointestinal (GI, 10.7%), and endocrine (10.1%) [6]. These irAEs are even more frequent and severe in patients treated with combination CTLA-4 and PD-1 inhibitors [6,7]. In the CheckMate 067 trial evaluating front-line ICI in metastatic melanoma, the rate of treatment-related grade 3–4 irAEs was 59% for those who received nivolumab and ipilimumab, compared with 28% and 23% for ipilimumab or nivolumab alone, respectively [8]. Notably, CTLA-4 and PD-1 inhibitors have been reported to exhibit different irAE profiles; colitis and hypophysitis are more common with the former, and pneumonitis and thyroid abnormalities are more common with the latter [9]. Further, CTLA-4 blockade-associated irAEs are dose-dependent with severe events observed with a 10 mg/kg dose and lower toxicity with ‘flip dose’ ipilimumab at 1 mg/kg and nivolumab 3 mg/kg [10,11,12]. Most irAEs can be managed effectively with discontinuation of the ICI, but may require corticosteroids or other immunosuppressive agents [13]. However, some irAEs can be permanent, life-threatening, or even fatal in <1% of patients [14]. High-grade irAEs generally necessitate hospitalization, intravenous corticosteroids, and the involvement of multidisciplinary specialists [13]. There is extensive research evaluating predictive biomarkers of response to ICIs [15,16]; however, outside of the presence of known underlying autoimmune disease or in the setting of organ transplant, there is a dearth of information predicting those who may be vulnerable to irAEs [17,18]. Limited studies have highlighted the potential impact of effector memory CD4^+^ T-cells at baseline, immune cells, increased T-cell receptor diversity, and the presence of auto-antibodies associated with heightened risk of irAEs [19], but very few studies have evaluated the impact of germline variants. 

The Personalized OncoGenomics (POG) program at BC Cancer seeks to align patients with advanced, metastatic cancer to data-informed treatment, using whole genome and transcriptome analysis (WGTA) of a fresh tumor biopsy [20,21]. Our group has previously shown that WGTA can help to identify factors that can predict immunotherapy efficacy [15]. We hypothesized that there may be underlying germline variants associated with the risk of developing irAEs. Herein, we evaluated all POG patients who received ICIs and developed irAEs to investigate whether there were any covariates or single nucleotide polymorphisms (SNPs) associated with irAEs.

## 2. Materials and Methods

### 2.1. Clinical Data Collection and Processing

All systemic therapies received by POG patients were abstracted from the BC Cancer Pharmacy database and supplemented by clinical chart review. ICI therapies were categorized as single-agent PD-1/PD-L1 inhibitor, combination CTLA-4/PD-1/PD-L1 inhibitor, single-agent CTLA-4 inhibitor, or other ICI (single or combination). ICIs were included as combination therapies if two different classes of agent were given simultaneously, or within 12 weeks of one another. The use of different classes of agents more than 12 weeks apart (n = 2) was included as “other single agents”. Some patients may have received chemotherapy in combination with ICIs through clinical trials. IrAEs were graded through retrospective chart review and grouped by organ system, using the framework provided by ASCO IrAE guidelines, which follows CTCAE 5.0 for most toxicities [13]. Clinical intervention was defined as use of PO/IV steroids +/− other immunomodulatory agents. Pre-existing autoimmune conditions were grouped as follows: Thyroid: Graves’ disease, hypothyroid; Gastrointestinal: Ulcerative colitis, Crohn’s disease; Musculoskeletal: Psoriatic arthritis, ankylosing spondylitis; Dermatological: Psoriasis, vitiligo; Hematological: Pernicious anemia, Immune thrombocytopenia (ITP); Other: Celiac disease.

### 2.2. Tissue Collection and Library Construction 

Tumor specimens were collected from biopsies or surgical resections, the pathology was reviewed, and nucleic acids were extracted as described previously [21]. Constitutional DNA representing normal cells was extracted from peripheral blood at the time of the biopsy. PCR-free DNA libraries and either strand-specific or ribodepleted RNA libraries were constructed [21].

### 2.3. Whole-Genome and Transcriptome Sequencing

Tumor genomes were sequenced to a target depth of 80× coverage and normal peripheral blood samples to 40× coverage on Illumina HiSeq 2500 using v3 or v4 chemistry and paired-end 125 base reads or on HiSeqX using v2.5 chemistry and paired-end 150 base reads, as described in detail in Pleasance et al. [21]. Transcriptomes were sequenced targeting 150–200 million 75-base paired-end reads on Illumina HiSeq2500 or on NextSeq500 using v2 chemistry. Sequences were aligned to human genome version hg19 using BWA v0.5.7 [22], and single-nucleotide variants (SNV) and insertions and deletions (indels) were called using Strelka v0.4.62 [23] and mutationSeq v1.0.2 [24].

### 2.4. HLA and Autoimmune-Associated SNPs

Patient MHC class I alleles were identified using Optitype (v1.3.1) [25]. Optitype was run on all three libraries (tumor DNA, tumor RNA, and constitutive DNA), and a consensus of all three was used. For cases that were discrepant between the libraries, manual review of the region determined the final alleles. Associations between HLA supertypes identified in at least five patients (n = 32) were calculated using Fisher’s exact test statistics and were multiple-test corrected using Holm–Bonferroni correction for 96 tests. 

The 25 SNPs that were examined in germline samples were selected from Chat et al. [26], and were also evaluated using Fisher’s exact statistics and were multiple-test corrected for 75 tests. 

Clinical and genomic data described for this cohort are made available.

### 2.5. Statistics

All statistical tests were performed using R version 3.6.3. Enrichment tests were performed using Fisher’s exact tests unless otherwise specified. *p*-values ≤ 0.05 were considered to be statistically significant, and q ≤ 0.05 where multiple test correction was used. *p* ≤ 0.10 and q ≤ 0.10 were considered to be trends.

## 3. Results

We identified 117 adult patients (≥18 years) enrolled in the POG program with a diagnosis of advanced or metastatic cancer who had been treated with an ICI during their treatment course (Table 1) [15]. Patients in this cohort either received an ICI prior (n = 60, 51%) or subsequent (n = 57, 49%) to the POG-directed biopsy. The median age was 58 years old at the time of ICI treatment (range: 27–86), and 55% (n = 64) of the cohort were female. Lung cancer was the most common disease type (24%, n = 28), followed by cutaneous melanoma (14%, n = 16), breast cancer (11% n = 13), and pancreatic cancer (11%, n = 13). 

Most patients received a single-agent PD-1 (n = 52)/PD-L1 inhibitor (n = 14, [total: 66, 56%], Table 1). In total, 32 patients (27%) received combination PD-1/PD-L1 + CTLA-4 inhibitors; 12 of which were in combination with chemotherapy. The remainder received single-agent CTLA-4 inhibitors (n = 2), NKG2A inhibitors (n = 2), PD-1/PD-L1 in combination with IDO-1 inhibitors (n = 10), multiple single-agent ICIs more than 12 weeks apart (n = 2), or other combination ICIs (PD-1/PD-L1 and OX-40 agonists, n = 3). Patients received ICIs for an average of 112 days (median, range: 1 [1 cycle]- 1674 days [4.5 years]). 

### 3.1. Distribution and Frequency of Immune-Related Adverse Events

**Figure 1:**
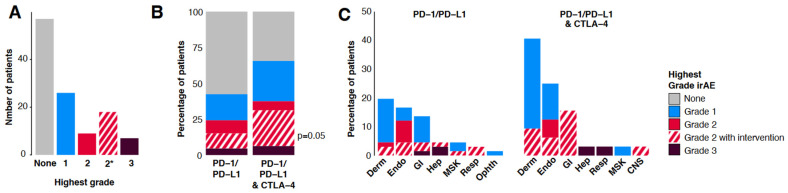
**Immune-related adverse event distribution:** (**A**). IrAEs documented in the cohort by grade. Grade 2* indicates a grade 2 irAE that required clinical intervention (Methods). (**B**). IrAEs by grade in patients treated with single and combination PD-1/PD-L1 agents, and in combination with CTLA-4. (**C**). IrAEs by grade and organ system in patients treated with single and combination PD-1/PD-L1 agents, and in combination with CTLA-4. Derm, dermatological; Endo, endocrine; GI, gastrointestinal; Hep, hepatic; MSK, musculoskeletal; Resp, respiratory; CNS, central nervous system; Ophth, ophthalmologic.

Sixty patients (51%) had a documented irAE (Methods, ASCO guidelines [13]) following treatment with an ICI (Figure 1A, Supplemental Table S1). Grade 1 (n = 26) and grade 2 (n = 27) irAEs occurred in 22% and 23% of patients, respectively; the latter included 18 patients (67%) who required corticosteroids, the majority of whom were systemic (n = 15). The remaining three received topical steroids only for dermatological conditions. Three patients received levothyroxine or methimazole for the management of thyroid-related conditions. Grade 3 irAEs occurred in seven patients (6%) and there were no grade 4 events. In total, 17 patients (28%) had permanent discontinuation of their ICI due to an irAE, and an additional 16 patients (27%) required a temporary hold of their ICI treatment due to the irAE. In the whole cohort, three patients (2.5%) were hospitalized for management of their irAE. 

The proportion of patients with irAEs was higher in those treated with combination PD-1/PD-L1 and CTLA-4 ICIs compared with single-agent PD-1/PD-L1 (66% vs. 42%, any grade irAE, *p* = 0.05, Figure 1B, Fisher’s exact test). Grade 3 irAEs occurred at a similar frequency in patients treated with single-agent PD-1/PD-L1 (n = 3, 4.5%, *p* = 0.66) or in combination with CTLA-4 (n = 2, 6.3%). It is of note that grade 3 irAEs were observed at a higher rate in patients treated with PD-1 inhibitors in combination with IDO-1 inhibitors (n = 2/10, 20%); however, the number of patients treated with this combination was low overall. 

The most common irAEs across the cohort were dermatological (n = 33, 28%, Figure 1C, Supplemental Figure S1A), in both PD-1/PD-L1-treated (19.7%, n = 13) and in combination with CTLA-4 (40.6%, n = 13). Almost all of these were low grade (n = 26 grade 1, 79%) and included rash/inflammatory dermatitis (n = 23, 88%), vitiligo (n = 2, 8%), and bullous dermatoses (n = 1). Endocrine and gastrointestinal (GI) related were the second and third most common irAEs in both PD-1/PD-L1 single-agent and combination with CTLA-4 treated groups (Figure 1C). In both treatment groups, colitis was the most common GI irAE and primary hypothyroidism was the most common endocrine irAE. Primary adrenal insufficiency and hypophysitis were both noted in single patients treated with combination PD-1/PD-L1 and CTLA-4-treated but not in patients treated using single-agent PD-1/PD-L1. 

The majority of grade 3 irAEs were hepatitis (n = 5/7, 71%, single-agent PD-1/PD-L1 n = 2; IDO-1 inhibitor, n = 2; combination PD-1 and CTLA-4 [single agents within 12 weeks of one another], n = 1), and the remaining two patients had colitis (single-agent PD-1) and pneumonitis (combination PD-L1 and CTLA-4). The majority (85.7%, n = 6/7) of grade 3 irAEs received steroid treatment. Overall, 28.6% of patients with grade 3 irAEs (n = 2/7) were hospitalized and 57.1% (n = 4/7) had permanent discontinuation of their ICI. The only patient with a grade 3 irAE (hepatitis [AST > 6 × upper limit of normal [ULN], ALT > 3 × ULN, ALP > 6 × ULN, GGT > 19 × ULN, bilirubin within normal limits]) who was not prescribed systemic steroids, was a patient with metastatic uveal melanoma treated with pembrolizumab. It was clinically unclear at the time if the patient was exhibiting drug-induced hepatitis or disease progression. The bloodwork was serially monitored and showed improvement in liver enzymes with cessation of pembrolizumab. The ICI was rechallenged successfully with waxing and waning liver enzymes throughout the treatment course of two years. This was likely consistent with immune-related cholangiopathy, given the predominant pattern of GGT elevation [27]. 

IrAE frequency was similar by age (Supplemental Figure S1B–D, ≥60 vs. <60, *p* = 0.46, any irAE; *p* = 1.0, grade 3 irAE), ethnicity (*p* = 0.85., any; *p* = 1.0, grade 3), and sex (*p* = 0.62, any; *p* = 0.26, grade 3) among patients treated with single-agent PD-1/PD-L1 inhibitors. Similarly, for combination PD-1/PD-L1 and CTLA-4 inhibitors, there was no association between the frequency of irAEs and age (*p* = 0.72, any irAE; *p* = 1.0, grade 3), ethnicity (*p* = 0.12, any; *p* = 1.0, grade 3), or sex (*p* = 0.27, any; *p* = 1.0, grade 3).

### 3.2. Co-Occurrence of irAEs

**Figure 2:**
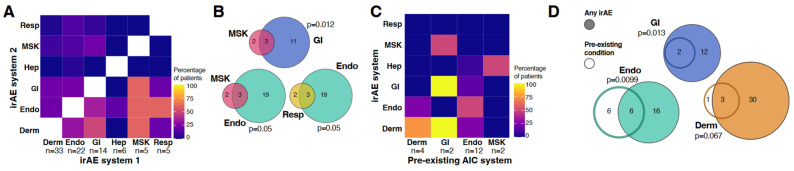
**Co-occurring immune-related adverse events (irAEs) and pre-existing autoimmune conditions:** (**A**). Co-occurring organ systems with irAEs. The primary system is listed horizontally (*x*-axis), and the proportion of patients with irAEs in this system having corresponding secondary irAE systems is shown. (**B**). Common co-occurring irAE systems. Intersect indicates the number of patients exhibiting an irAE of each system. (**C**). Co-occurring organ systems between autoimmune conditions and irAEs. The proportion of patients with autoimmune conditions who have an irAE in each system is shown vertically. (**D**). Overlap between pre-existing autoimmune conditions and irAEs. *p*-values are derived from Fisher-exact statistics. Derm, dermatological; Thyroid; GI, gastrointestinal; Hep, hepatic; MSK, musculoskeletal; Resp, respiratory; CNS, central nervous system; Ophth, ophthalmologic. Organ systems with less than three observations (Ophth, CNS) are not shown in (**A**,**C**).

Most irAEs were confined to a single organ system (n = 42, 70%); however, almost a third of patients with irAEs (30%, n = 18) experienced irAEs in two or more different systems (Figure 2A). We did not observe a difference in the frequency of irAEs involving multiple organ systems between patients who received PD-1/PD-L1 inhibitors (35.7%, *p* = 0.53) and those with combination CTLA-4 (23.8%). Two patients had irAEs across four different systems, both of which included colitis (GI) and inflammatory dermatitis (derm). One patient with melanoma treated with nivolumab and ipilimumab additionally had pneumonitis (resp) and hyperthyroidism (endo), while the other, a patient with lung adenocarcinoma treated with nivolumab, had hypophysitis (endo) and aseptic meningitis (CNS). There was no association with multiple irAE organ systems and age (*p* = 1.0), sex (*p* = 0.40), or tumor type (*p* = 0.25), and there was no difference between single-agent and combination therapy (results not shown). 

When examining the co-occurrence of irAEs across different organ systems, we included all treatment types together to have enough power to determine associations. We found that irAEs in some organ systems were more likely to present together than others (Figure 2A). Patients with an MSK irAE (inflammatory arthritis, polymyalgia-like syndrome, or myositis) often also had a GI irAE (Figure 2B, *p* = 0.012, Fisher’s exact; colitis), or an endocrine toxicity (Figure 2B, *p* = 0.05; hypothyroidism). The combination of GI (colitis) and MSK (arthritis and polymyalgia-like syndrome) irAEs was observed in diverse malignancies (sarcomatoid carcinoma of the lung, pancreatic cancer, and gastric cancer) and treatment types (single-agent PD-1, combination PD-L1 and CTLA-4 in combination with chemotherapy, and two different single-agent PD-1 inhibitors [funding related]). Patients with both MSK and endocrine irAEs had squamous cell carcinoma of the tongue, gastric cancer, and high-grade serous ovarian cancer, and were treated with avelumab in combination with an OX-40 agonist, PD-1 inhibitor, pembrolizumab then nivolumab, and single-agent avelumab, respectively. Similarly, patients with a respiratory irAE (pneumonitis) often had an endocrine reaction (Figure 2C, *p* = 0.05; hypothyroidism, hyperthyroidism, or hypophysitis). The patient exhibiting hypophysitis had metastatic melanoma and had received combination nivolumab and ipilimumab, while the other two patients (lung adenocarcinoma and lung squamous cell carcinoma) both received single-agent PD-1 inhibitors (nivolumab/pembrolizumab).

### 3.3. Pre-Existing Autoimmune Conditions Correlate with irAEs

Twenty patients (17%) had at least one pre-existing autoimmune condition and in this group, there was a trend toward a higher likelihood of developing an irAE (*p* = 0.086, 70% vs. 47%, Fisher’s exact), including grade 3 (*p* = 0.096, grade 3, 15% vs. 4.1%). Of the three patients who were hospitalized, two had pre-existing autoimmune conditions (10% of patients with pre-existing conditions vs. 1% without, *p* = 0.075).

Pre-existing endocrine conditions (hypothyroidism, Graves’) were the most common pre-existing autoimmune conditions across the cohort (Table 1, 60%, n = 12), most often hypothyroidism (n = 11). Dermatological conditions, including psoriasis and vitiligo, were present in four patients, and pre-existing GI conditions were present in two patients (Crohn’s disease and ulcerative colitis). Patients with a pre-existing endocrine condition often had exacerbation of their condition (Figure 2C,D, *p* = 0.0099, n = 6, 50% of patients). This was similarly true for patients (both treated with nivolumab) with GI conditions (Crohn’s and ulcerative colitis) exhibiting GI irAEs, specifically colitis (Figure 2C,D, *p* = 0.013, n = 2, 100%). Patients with dermatological conditions (specifically psoriasis and treated with single-agent PD-1 inhibitors) trended toward an increased risk of developing a dermatological irAE (Figure 2C,D, *p* = 0.067, 75%). Interestingly, the two patients with pre-existing GI autoimmune conditions also exhibited dermatological irAEs (inflammatory dermatitis, grade 1 and 2, Figure 2C, *p* = 0.078).

### 3.4. Impact of Germline Factors on the Frequency and Type of IRAEs 

Previous studies have highlighted associations between germline SNPs and autoimmune conditions [26,28], including variation in the major histocompatibility complex (MHC) class I alleles and single nucleotide polymorphisms (SNPs). Thus, we explored whether germline variation in HLAs corresponding with MHC class I may be associated with an elevated risk of developing irAEs.

**Figure 3:**
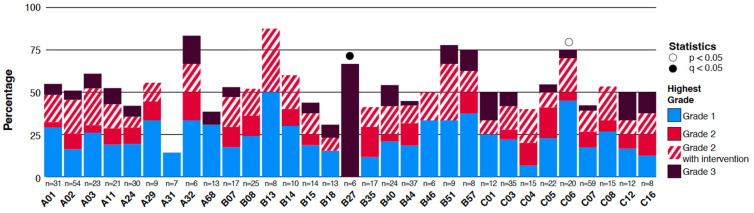
**Immune-related adverse events and HLA types:** Proportion of different grade irAEs associated with MHC class I supertypes detected in at least five patients. *p*-values are derived from Fisher-exact statistics.

The relationship between each of the 32 most common class I MHC supertypes present in our cohort and the occurrence and severity of irAEs was analyzed. We identified one supertype, HLA-B27, which was significantly associated with the development of grade 3 irAEs (Figure 3A, q = 0.007). Of the six patients presenting with this HLA-B27 supertype, representing 5% of all patients, 67% (n = 4/6) developed grade 3 irAEs (Supplemental Table S2), specifically hepatitis (n = 3) and pneumonitis (n = 1). In comparison, the frequency of grade 3 irAEs in HLA-B27 negative patients was only 2.7%. The patients with HLA-B7 and irAEs all required steroid intervention and one required hospitalization (pneumonitis). Two patients had received a combination PD-1/PD-L1 and CTLA-4 inhibitor therapy (durvalumab and tremelimumab, n = 1; nivolumab and ipilimumab, n = 1) while the other two received nivolumab with an IDO-1 inhibitor. The remaining two patients (33%) with HLA-B27 did not develop any irAEs and were treated with single-agent PD-1 inhibitors. 

Of the six patients in our cohort with an HLA-B27 allele, the most commonly observed subtype was HLA-B27:05 (n = 4), and the remaining two patients had HLA-B27:02 and B27:04, respectively. The patient with HLA-B27:04 had a pre-existing history of ankylosing spondylitis and developed grade 3 hepatitis on nivolumab with an IDO-1 inhibitor. 

The presence of HLA-C06 demonstrated a trend toward an association with the development of any irAE (*p* = 0.026), where 75% of patients with this allele had an irAE (n = 15/20), but this did not remain statistically significant after multiple-test correction (q = 1, Figure 3A, Supplemental Table S2). In this group, irAEs occurred following a variety of ICIs with 47% (n = 7) having received a single-agent PD-1/PD-L1 inhibitor. 

Germline polymorphisms linked to autoimmune conditions have been reported, and some (e.g., rs17388568) have also been associated with response to ICIs [26]. We measured the incidence of 25 SNPs previously linked to autoimmune conditions (Methods) in our cohort to determine if any were associated with irAEs (Supplemental Table S3). Several of these SNPs were common in our cohort (9–99% of patients) but were not associated with the development of irAEs (q < 0.05). 

## 4. Discussion

ICIs have changed the treatment landscape for many cancers, and with the success observed in advanced cancers, they are increasingly used in earlier stages of the disease. However, some patients develop irAEs that can be life-threatening, or cause significant morbidity. This is particularly true for combination therapy. Of note, the present study did not highlight the elevated risk of iRAEs with combination ipilimumab and nivolumab compared to single-agent PD1/PDL inhibitors: however, this cohort is selected for those with advanced recurrent disease. Patients treated with combination ICI who exhibit high-grade irAEs often also have durable remissions and likely would not have been enrolled in this study. Nevertheless, our cohort with uniformly collected blood allowed us to explore germline mutations associated with irAE across a broad range of cancers treated with ICI. Very few prior studies have explored factors that may predispose patients to irAEs, and whether there are host factors that may be associated with an elevated risk. To this end, we found that the inherited HLA type (HLA-B27) was associated with an increased risk of severe (grade 3) irAEs. 

Previous studies have demonstrated the association of the HLA-B27 supertype with ankylosing spondylitis [28], and a prior case report described PD-L1-induced encephalitis [29] in a patient with HLA-B27:05. However, our study suggests a link more broadly to high-grade irAEs for single-agent PD-1/PD-L1 inhibitors, as well as combination PD-1 and CTLA-4 inhibitors. We also noted the elevated risk in PD-1/PD-L1 inhibitors in combination with IDO-1 inhibitors. IDO inhibitors inhibit IDO (indoleamine 2,3-dioxgenase), an enzyme catabolizing tryptophan to kynurenine, which creates an immunosuppressive environment [30]. Phase 3 studies in metastatic melanoma were initiated by combining the IDO1 inhibitor epacodostat with pembrolizumab: however, there was no efficacy advantage [31]. As we are limited by small patient numbers, it may be that the baseline risk observed with IDO inhibitor combinations is driven by the PD-1 inhibitor, and is then enhanced in those with HLA-B27 supertype. 

Class I MHC alleles are essential for neoantigen presentation, and other genotypes (HLA-A*01, HLA-B*44, HLA-B*62, and heterozygosity of the class I loci) have been previously linked to the ICI response [32,33]. There are emerging data that certain HLA types may be associated with specific irAEs as described by Jiang et al.; however, that report mainly described cytopenias, which can be impacted by concurrent cytotoxic treatments that are often administered with ICIs [34]. Therefore, evaluating the potential benefit versus toxicity risk of certain alleles may be increasingly useful for predicting both clinical response and adverse reactions for ICI-treated patients.

Female sex is also an increasingly recognized factor implicated in the risk of severe adverse events, particularly with immunotherapy. A recent large study of clinical trial patients (PD-1/PD-L1 and combination with CTLA-4) showed an increased risk of severe toxicity in females compared to males (56.6% vs. 48.8%, OR 1.49, *p* < 0.001). The reasons for this remain unclear but may include underlying differences in pharmacokinetics and pharmacodynamics, and gut microbiome [35]. This pattern is also reflected in our cohort for grade 3 irAEs (9.4% vs. 1.9%, *p* = 0.13), but it is limited by small patient numbers and did not reach statistical significance. 

Over two-thirds of patients in our cohort with an underlying autoimmune condition prior to starting ICI exhibited irAEs, in line with other recent reports [36]. Overall, irAE events were numerically higher but ultimately only 15% of patients discontinued therapy as a result of irAEs compared to 3% who did not have a known underlying condition. This is comparable to a discontinuation rate of 8% in a recent study of 123 non-small cell lung cancer and melanoma patients with autoimmune disorders treated with a variety of ICIs [36]. ICIs represent an important treatment option for many patients including those with underlying autoimmune disease; however, this is currently an area under active study in a multicenter phase 1b trial evaluating the safety and efficacy of nivolumab in a large cohort of patients with autoimmune conditions (NCT03816345). 

Overall, despite some limitations to our study, including a selected cohort, small sample size, and lack of dosing information for all patients, we were able to identify factors associated with irAEs. Importantly, those with the HLA-B27 supertype represent a minority of patients (~6% of the US population [37]), but they do have a significantly elevated risk of high-grade irAEs. Ideally, screening may guide the choice of ICI, especially with the significant risks associated with combination therapy. Whole-genome sequencing paired with clinical follow-up through programs such as POG [20,21] offers rich resources in which to study biomarkers for ICI response and toxicity. 

## Figures and Tables

**Table 1:** Cohort characteristics and association with irAEs.

	N (%)		Any irAE (%)		Grade 2 Intervention (%)		Grade 3+ (%)	
**Tumor type**								
Lung	28	(23.9)	8	(28.6)	5	(17.9)	1	(3.6)
Melanoma (cutaneous)	15	(12.8)	9	(60)	5	(33.3)	0	(0)
Breast	13	(11.1)	7	(53.8)	3	(23.1)	1	(7.7)
Pancreatic	13	(11.1)	8	(61.5)	2	(15.4)	0	(0)
Gynecological	8	(6.8)	4	(50)	1	(12.5)	1	(12.5)
Melanoma (uveal)	8	(6.8)	7	(87.5)	3	(37.5)	3	(37.5)
Head and neck	7	(6.0)	3	(42.9)	1	(14.3)	0	(0)
Colorectal	6	(5.1)	2	(33.3)	1	(16.7)	0	(0)
Sarcoma	6	(5.1)	4	(66.7)	1	(16.7)	0	(0)
Cholangiocarcinoma	3	(2.6)	1	(33.3)	1	(33.3)	0	(0)
Gastric	3	(2.6)	2	(66.7)	1	(33.3)	0	(0)
Kidney	2	(1.7)	2	(100)	0	(0)	0	(0)
Thymoma	2	(1.7)	1	(50)	0	(0)	0	(0)
Adenoid cystic carcinoma	1	(0.9)	0	(0)	0	(0)	0	(0)
Adrenocortical	1	(0.9)	1	(100)	1	(100)	1	(100)
Lymphoma	1	(0.9)	0	(0)	0	(0)	0	(0)
Testicular	1	(0.9)	1	(100)	0	(0)	0	(0)
**Age**								
≥60 years	56	(47.9)	26	(46.4)	8	(14.3)	2	(3.6)
<60 years	61	(52.1)	34	(55.7)	17	(27.9)	5	(8.2)
**Sex**								
Female	64	(54.7)	33	(51.6)	15	(23.4)	6	(9.4)
Male	53	(45.3)	27	(50.9)	10	(18.9)	1	(1.9)
**Pre-existing autoimmune condition**								
Yes	20	(17.1)	14	(70)	5	(25)	3	(15)
No	97	(82.9)	46	(47.4)	20	(20.6)	4	(4.1)
**Pre-existing autoimmune condition**								
Thyroid disease	12	(10.3)	9	(75)	3	(25)	2	(16.7)
Gastrointestinal	2	(1.7)	2	(100)	2	(100)	0	(0)
Musculoskeletal	2	(1.7)	1	(50)	1	(50)	1	(50)
Dermatological	4	(3.4)	3	(75)	0	(0)	0	(0)
Hematological	1	(0.9)	0	(0)	0	(0)	0	(0)
Other	2	(1.7)	1	(50)	0	(0)	0	(0)
**ICI inhibitor type**								
Single-agent PD-1/PD-L1	66	(56.4)	28	(42.4)	10	(15.2)	3	(4.5)
Combination PD-1/PD-L1 & CTLA-4	32	(27.4)	21	(65.6)	10	(31.3)	2	(6.3)
Single-agent CTLA-4	2	(1.7)	2	(100)	0	(0)	0	(0)
Other *	17	(14.5)	9	(52.9)	5	(29.4)	2	(11.8)

* Other includes PD-1/PD-L1 in combination with IDO-1 inhibitors (n = 10), NKG2A inhibitors (n = 2), single-agent ICIs received more than 12 weeks apart (n = 2), PD-1/PD-L1 inhibitors in combination with OX-40 agonists (n = 3). Autoimmune conditions are grouped as follows: Thyroid—Graves’ (n = 1), hypothyroid (n = 11); Gastrointestinal—Crohn’s (n = 1), ulcerative colitis (n = 1); Musculoskeletal—psoriatic arthritis (n = 1), ankylosing spondylitis (n = 1); Dermatological—psoriasis (n = 3), vitiligo (n = 1); Hematological—Immune thrombocytopenia; Other—celiac disease (n = 1), pernicious anemia (n = 1).

## Data Availability

Data on mutations, copy changes, and expression organized by tumor type are available for samples in the POG program from https://www.personalizedoncogenomics.org/cbioportal/. Previously published data (RNA and WGS) for immunotherapy-treated POG patients (Pender et al., [15]) are available from EGA under the study EGAS00001001159. All other data from this study may be available from the corresponding author on reasonable request.

## Supplementary Materials

The following supporting information can be downloaded at: https://www.mdpi.com/article/10.3390/curroncol31040140/s1, Supplemental Figure S1. A. IrAEs by grade and organ system in all patients treated with ICIs. B–D. IrAEs by grade and age group (B), ethnicity (C), and sex (D) in patients treated with single and combination PD-1/PD-L1 agents, and in combination with CTLA-4. Supplemental Table S1. Full patient characteristics, treatments received, and irAEs. Supplemental Table S2. Associations between ICI therapy and patient HLA alleles. Supplemental Table S3. Associations between ICI therapy and germline SNPs.
