# Supplementary material for: Exploration of Germline Correlates and Risk of Immune-Related Adverse Events in Advanced Cancer Patients Treated with Immune Checkpoint Inhibitors
Source: Curr Oncol. 2024 Mar 30;31(4):1865–75. doi: 10.3390/curroncol31040140 (PMC11048877; doi:10.3390/curroncol31040140)
Supplement: Supplementary file 1 [file curroncol-31-00140-s001.zip › Sup_figure1.pdf]

Supplemental Figure 1

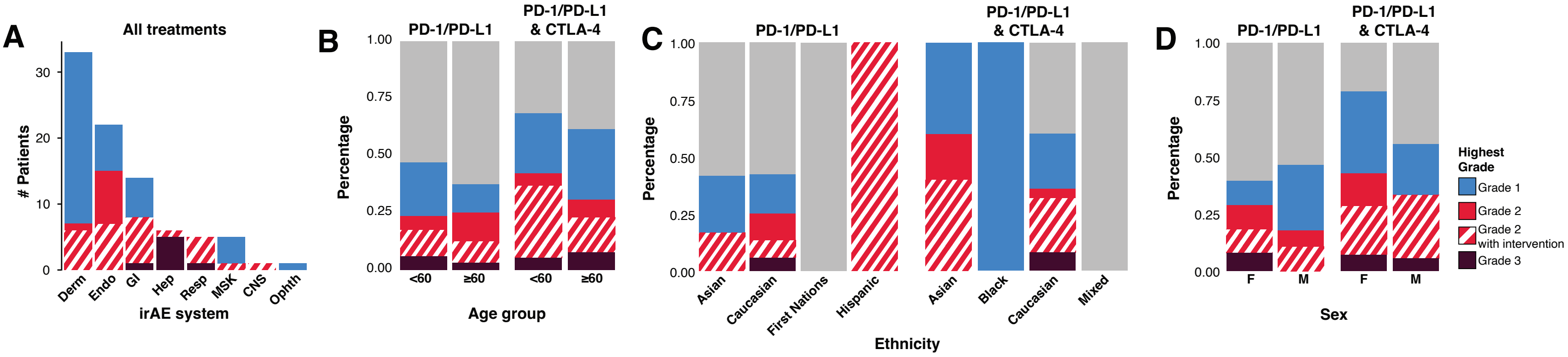

Supplemental Figure 1. A. IrAEs by grade and organ system in all patients treated with ICIs. B-D. IrAEs by grade and age group (B), ethnicity (C) and sex (D) in patients treated with single and combination PD-1/PD-L1 agents, and in combination with CTLA-4.
